# Essential Public Health Functions in Ireland: Perspectives to strengthen capacities and stewardship

**DOI:** 10.1093/eurpub/ckac129.207

**Published:** 2022-10-25

**Authors:** G McDarby, S Mustafa, Y Zhang, R Seifieldin, S Saikat, L Hendricks, T McNicholas

**Affiliations:** 1 Health Services Resilience Team, WHO, Geneva, Switzerland; 2 Department of Health, Government of Ireland, Dublin, Ireland

## Abstract

**Background:**

COVID-19 has caused unprecedented disruptions to health, social and economic systems in countries worldwide including Ireland. Weaknesses in Public Health capacities have undermined health system resilience compounding the effects of the pandemic. The Essential Public Health Functions (EPHFs) provide a comprehensive, cost-effective approach to operationalising public health and a means to build health systems resilience. As Ireland looks to recovery, the Department of Health engaged the World Health Organization (WHO) to undertake a mapping of the current state of delivery of EPHFs to identify opportunities for improvement and support wider health system strengthening towards resilience.

**Methods:**

A strategic review of the delivery of EPHFs in Ireland was conducted with respect to policy, infrastructure, service provision and coordination and integration. Findings were reported in the context of international lessons identified through experience with COVID-19 and major health system challenges within the Irish context.

**Results:**

There are significant capacities present within the Irish context to support the delivery of the EPHFs though they are limited in strategic cohesion, coordination and implementation. These include a high level of Public Health expertise, an agile and resourceful workforce, a strongly engaged community and significant evidence generation and synthesis capacities. Gaps recognised included ICT infrastructure and capacity, workforce resourcing and support, pandemic planning and public health governance, visibility, legislation, strategy and resourcing. COVID-19 has led to the development and strengthening of mechanisms to leverage a whole-of-government and -society approach to health that should be sustained to tackle ongoing and future stressors.

**Conclusions:**

The use of the EPHFs within the Irish setting provides a comprehensive approach to strengthening capacities for public health and enhanced population health and wellbeing.

